# College Students’ Employability, Cognition, and Demands for ChatGPT in the AI Era Among Chinese Nursing Students: Web-Based Survey

**DOI:** 10.2196/50413

**Published:** 2023-12-22

**Authors:** Yuanyuan Luo, Huiting Weng, Li Yang, Ziwei Ding, Qin Wang

**Affiliations:** 1 Clinical Nursing Teaching and Research Section The Second Xiangya Hospital of Central South University Changsha China; 2 Xiangya School of Nursing Central South University Changsha, Hunan China

**Keywords:** college students’ employability, artificial intelligence quotient, ChatGPT, nursing students, China, college student, AI, artificial intelligence

## Abstract

**Background:**

With the rapid development of artificial intelligence (AI) and the widespread use of ChatGPT, nursing students’ artificial intelligence quotient (AIQ), employability, cognition, and demand for ChatGPT are worthy of attention.

**Objective:**

We aimed to investigate Chinese nursing students’ AIQ and employability status as well as their cognition and demand for the latest AI tool—ChatGPT. This study was conducted to guide future initiatives in nursing intelligence education and to improve the employability of nursing students.

**Methods:**

We used a cross-sectional survey to understand nursing college students’ AIQ, employability, cognition, and demand for ChatGPT. Using correlation analysis and multiple hierarchical regression analysis, we explored the relevant factors in the employability of nursing college students.

**Results:**

In this study, out of 1788 students, 1453 (81.30%) had not used ChatGPT, and 1170 (65.40%) had never heard of ChatGPT before this survey. College students’ employability scores were positively correlated with AIQ, self-regulation ability, and their home location and negatively correlated with school level. Additionally, men scored higher on college students’ employability compared to women. Furthermore, 76.5% of the variance was explained by the multiple hierarchical regression model for predicting college students’ employability scores.

**Conclusions:**

Chinese nursing students have limited familiarity and experience with ChatGPT, while their AIQ remains intermediate. Thus, educators should pay more attention to cultivating nursing students’ AIQ and self-regulation ability to enhance their employability. Employability, especially for female students, those from rural backgrounds, and students in key colleges, deserves more attention in future educational efforts.

## Introduction

### Overview

Artificial intelligence (AI) refers to using computer technology to simulate the operating mechanisms of the human brain, thus enabling computer applications in vision, speech recognition, and natural language processing. It aims to simulate and extend the human thought, learning, and knowledge storage process [1,2]. In November 2022, ChatGPT, a generative AI tool, was officially launched. By January 2023, ChatGPT had more than 100 million registered users, making it the fastest-growing consumer application to date, further advancing human society into an intelligent stage and quickly becoming the focus of attention and discussion in education [3]. As the field of AI develops and is implemented globally, it has brought great convenience and economic benefits to human society, impacting the roles within it [4,5]. Whether AI will replace some industries in the future has also become a topic of intense debate [6-8]. In recent years, the application of AI in nursing has gradually expanded [9]. Studies have pointed out that the benefits and potential applications of ChatGPT in health care education research and practice include personalized medicine, prediction of disease risk and outcome, streamlining the clinical workflow, improved diagnostics, facilitating high-quality text writing, accelerating literature reviews, improving the personalized learning experience, and being an adjunct in group learning. However, it also runs the risk of moral hazard, plagiarism, and misinformation dissemination [10-12]. In the information age, the ability of nursing students to cope with this impact and sieze opportunities is very important for their future studies and work. 

Zuobing Wang [13] first proposed artificial intelligence quotient (AIQ) in his book The Education Revolution in the Age of Artificial Intelligence. He pointed out that AIQ is “the ability of human beings to use artificial intelligence technology” [13]. According to him, different times have different requirements for human workers, and the AI era requires human workers to have AIQ [13]. Polson Nick [14] also developed this concept, pointing out that AIQ combines artificial and human intelligence. Only by realizing the integration of humans and intelligence can humans and machines work more intelligently [14]. Notably, college student employability refers to the skills college students need to develop to achieve employment [15]. For college students, employability refers to their ability to secure and maintain a job that aligns with their education after graduation [16,17]. Self-regulation is an important part of Bandura’s social learning theory [18]. This is a process through which individuals regulate their behavior, cognition, and emotion to meet the expected standards set by themselves. Human beings can pursue the achievement of goals and align themselves with the environment through self-regulation [19]. Furthermore, a study showed that self-regulation could affect college students’ employability [20]. However, there are few studies on AIQ and self-regulation of nursing students and even less research on their relationship with nursing students’ employability in China and abroad. In the context of the rapid development of AI, information technology is being increasingly used in nursing. Studies have also found that nurses’ nursing information capabilities are closely related to patients’ sense of safety, clinical nursing system use, and scientific research capabilities, affecting patient health outcomes [21]. Moreover, improving nurses’ nursing information ability can also help with making clinical decisions, improving nursing quality, promoting nurses’ self-development, and improving their sense of professional benefits [22-24]. Additional studies have also shown that the use of AI in teaching nursing has received overall positive evaluations from nursing students, confirming AI’s acceptability, feasibility, and usability in the field of nursing education [25,26]. As the main force of the nursing profession, nursing students deserve attention concerning their ability to use AI, their capacity for self-adjustment to adapt to clinical nursing work in the information age, and their employability. 

Therefore, we conducted a web-based survey in China to investigate the cognition and demand for the latest AI tool—ChatGPT. We also examined the AIQ status of nursing students in this particular era to analyze the correlation between AIQ and the employability of nursing college students. Additionally, we analyzed the factors associated with college students’ employability to guide future initiatives in nursing intelligence education and improve the employability of nursing students.

### Objective

This study aimed to investigate Chinese nursing students’ cognition and demand for the latest AI tool ChatGPT and their AIQ status to provide directions for future intelligent nursing education in the era of AI. Additionally, it aimed to analyze the correlation between AIQ and the employability of nursing college students as well as the factors associated with college students’ employability.

## Methods

### Design

This cross-sectional study was conducted from March 21, 2023, to April 25, 2023. This study adopted a convenience sampling and snowballing method, and the study participants were recruited through the internet and sent open questionnaires through Sojump (a program developed by Changsha Xingxin Information Technology Company for collecting questionnaires). Our entire questionnaire had a total of 53 items, and the sample size was 10-20 times that of the questionnaire items. Considering a 10% allowance for lost follow-up and invalid questionnaires, the recommended sample size was 583-1166.

Inclusion criteria were as follows: (1) students currently enrolled in nursing and (2) voluntary participation in this study. Exclusion criteria included those with barriers to questionnaire completion.

We configured the corresponding settings in Sojump. As such, the same IP address was only allowed to fill out the questionnaire once to ensure that each participant represented a unique entry. Additionally, we set up a questionnaire completeness check. Finally, the data of 1788 nursing students from 30 provincial-level administrative regions in China were collected for this study. Before the questionnaire was filled out, we introduced the purpose of the study. We then asked the participants to carefully fill out the questionnaire, which was filled out voluntarily without any compensation.

### Measurements

#### General Information Questionnaire

A general information questionnaire developed by our research group was used. General information included age (coded 1-5 for “younger than 18 years,” “18-21 years,” “22-25 years,” “26-30 years,” and “older than 30 years,” respectively), gender (coded 1 for “male” and 2 for “female”), school level (coded 1-3 for ‘‘junior college,” “common undergraduate course college,” and “high-level university,” respectively), degree (coded 1-3 for “associated degree or below,” “baccalaureate degree,” and “master’s degree or above,” respectively) and home location (coded 1-3 for “countryside,” “counties and towns,” or “cities,” respectively).

#### “AIQ–Self-Regulation–College Students’ Employability” Questionnaire

The “AIQ–Self-Regulation–College Student Employability” questionnaire was developed by Yuhan Wang [20] in 2021 and consisted of 3 questionnaires: AIQ, Self-Regulation, and College Students’ Employability, using a 5-point Likert scale. The AIQ questionnaire includes 4 dimensions: creativity power (CP), data power (DP), communication ability (CA), and learning ability (LA). The self-regulation questionnaire includes 2 dimensions: the ability of individual recognition (AIC) and the ability of individual acceptance (AIA). College students’ employability includes 4 dimensions: career support skills, general vocational skills, competitive skills, and individual traits. The reliability of the questionnaire was good. Furthermore, the Cronbach α coefficients of the 3 subquestionnaires were 0.907, 0.877, and 0.926, respectively.

#### AI Awareness-Demand Questionnaire

Our research group compiled the AI Awareness-Demand questionnaire. The expert group comprised 5 nursing educators and 5 clinical nursing staff members. Furthermore, the questionnaire collected their views, attitudes, and needs for AI and the latest AI tool—ChatGPT.

### Data Analysis

Descriptive statistics were presented as numbers and percentages for demographic information and students’ cognition and demand for ChatGPT. After conducting the normality test on the employability score of college students, if the normal distribution was satisfied, independent samples *t* tests (2-tailed) and one-way ANOVAs were used to compare differences in college students’ employability scores based on sample characteristics. If normal distribution assumptions were not met, nonparametric tests were used. Pearson correlation tests were used to explore the relationship between college students’ employability, AIQ, and self-regulation scores. We used the direct input method. Variables related to employability in Pearson correlation analysis and demographics variables with a *P*<.05 were entered into a multiple hierarchical regression analysis to assess the relationship between college students’ employability scores and AIQ scores, self-regulation scores, and demographics. Furthermore, we used SPSS for macOS (version 24.0; IBM Corp) to perform all statistical analyses.

### Ethical Considerations

Our questionnaires were filled out anonymously, and we guaranteed that all participants’ private information was kept confidential. All participants were informed and consented to participate in this study without any compensation, and we requested informed consent from their guardians for participants aged <18 years. This study was approved by the Ethics Review Committee for Nursing and Behavioral Medicine Research, School of Nursing, Central South University (ID E202333).

## Results

### Sample Characteristics

**Figure 1:**
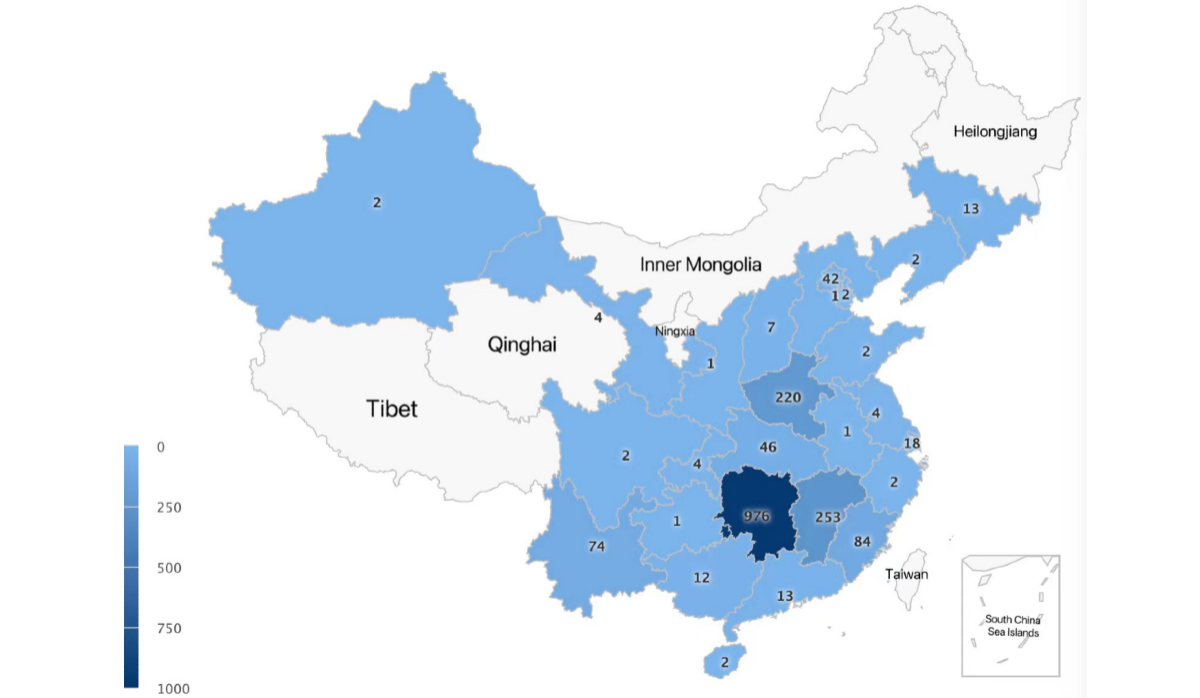
Distribution of the respondents.

A total of 1788 nursing students from 30 provincial-level administrative regions in China (Figure 1) eventually responded to this study. The respondents’ sociodemographic characteristics are shown in Table 1.

**Table 1:** The sociodemographic characteristics of the respondents (N=1788).

Characteristics		Values, n (%)
**Gender**		
	Male	234 (13.10)
	Female	1554 (86.90)
**Age (years)**		
	<18	44 (2.50)
	18-21	1490 (83.30)
	22-25	231 (12.90)
	26-30	14 (0.80)
	>30	9 (0.50)
**School level^a^**		
	High-level university (eg, “Double First Class” universities, “985,” and “211”)	97 (5.40)
	Common undergraduate course college	813 (45.50)
	Junior college	878 (49.10)
**Degree**		
	Master’s degree or higher	77 (4.30)
	Baccalaureate degree	869 (48.60)
	Associate’s degree or lower	842 (47.10)
**Location of home**		
	City	343 (19.20)
	Counties and towns	422 (23.60)
	Countryside	1023 (57.20)

^a^According to China’s division of university levels, some high-level universities are included in national initiatives or projects called the “Double First-Class” initiative, “Project 985,” and “Project 211.”

### Chinese Nursing Students' Cognition and Demand for the Latest AI Tool (ChatGPT)

**Figure 2:**
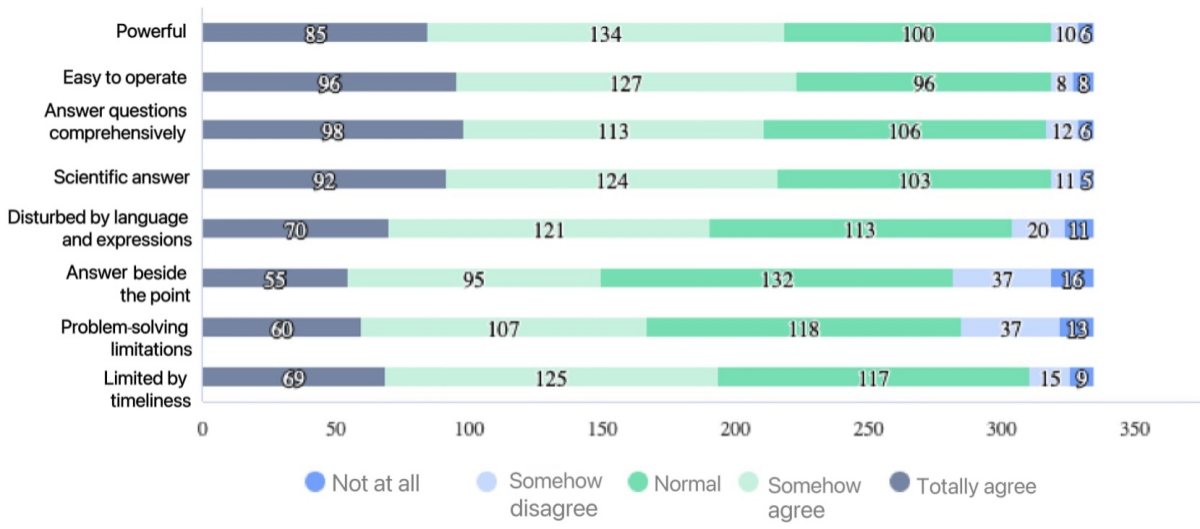
The user experience of those who have used ChatGPT.

In this study, 1453 (81.30%) students had not used ChatGPT, and 1170 (65.40%) had never heard of ChatGPT before this survey. Additionally, 357 (19.97%) and 376 (21.03%) students believed AI affected their major and future employment, respectively. The average AIQ score was 41.28 (SD 8.68), which was at a medium level. More details are shown in Table 2. Moreover, the user experience of those who have used ChatGPT among the respondents is presented in Figure 2.

**Table 2:** Chinese nursing students’ cognition and demand for the artificial intelligence (AI) tool (ChatGPT).

Question			Values
**Have you ever heard of ChatGPT? (N=1788), n (%)**			
	Yes	618 (34.60)	
	No	1170 (65.40)	
**Ways to know ChatGPT (n=618), n (%)**			
	Friends recommend	157 (25.40)	
	Social software (eg, WeChat and QQ)	154 (24.92)	
	Online apps (eg, MicroBlog and TikTok)	206 (33.33)	
	Search engines (eg, Baidu and Google)	78 (12.62)	
	Other	23 (3.72)	
**Whether you have used ChatGPT (N=1788), n (%)**			
	Yes	335 (18.70)	
	No	1453 (81.30)	
**Reason for not using ChatGPT (n=1453), n (%)**			
	I don’t need to use ChatGPT	379 (26.08)	
	I can’t use ChatGPT due to the account and network	106 (7.30)	
	I don’t know how to access ChatGPT	485 (33.38)	
	I don’t know how to use ChatGPT	226 (15.55)	
	I am not sure if the answer provided by ChatGPT is correct	75 (5.16)	
	Fear of privacy disclosure	120 (8.26)	
	Other	62 (4.27)	
**Feeling of using ChatGPT (n=335), n (%)**			
	Very dissatisfied	10 (2.99)	
	Dissatisfied	12 (3.58)	
	Normal	103 (30.75)	
	Satisfied	129 (38.51)	
	Very satisfied	81 (24.18)	
**Acceptance of AI** **(N=1788), n (%)**			
	Very willing to accept	551 (30.82)	
	Willing to accept	814 (45.53)	
	Normal	378 (21.14)	
	Harder to accept	22 (1.23)	
	Totally unacceptable	23 (1.29)	
**Do you think AI will have an impact on your major? (N=1788), n (%)**			
	No	1431 (80.03)	
	Yes	357 (19.97)	
**Do you think AI will have an impact on your future employment? (N=1788), n (%)**			
	No	1412 (78.97)	
	Yes	376 (21.03)	
Artificial intelligence quotient score, mean (SD)			41.28 (8.68)
Creativity power, mean (SD)			10.63 (2.47)
Data power, mean (SD)			9.83 (2.52)
Communication ability, mean (SD)			10.33 (2.23)
Learning ability, mean (SD)			10.48 (2.42)

### The Bivariate Analysis

According to the bivariate analysis, college students’ employability is related to gender, age, school level, degree, and home location (*P*<.05). The results of the bivariate analysis are presented in Table 3.

**Table 3:** The results of the bivariate analysis (N=1788).

Characteristics			College students’ employability, mean (SD)		*P* value	
**Gender**						.01
	Male	52.92 (13.98)				
	Female	50.49 (10.15)				
**Age (years)**						.007
	<18	50.57 (13.47)				
	18-21	50.82 (10.97)				
	22-25	50.34 (8.30)				
	26-30	51.86 (11.45)				
	>30	60.33 (11.53)				
**School level**						<.001
	High-level university	54.59 (12.69)				
	common undergraduate course college	50.33 (9.41)				
	Junior college	50.83 (11.59)				
**Degree**						<.001
	Master’s degree or higher	56.52 (13.50)				
	Baccalaureate degree	50.57 (9.43)				
	Associate’s degree or lower	50.54 (11.60)				
**Location of home**						.005
	City	53.24 (11.28)				
	Counties and towns	50.28 (11.16)				
	Countryside	50.21 (10.29)				

### Correlation Analysis of Influencing Factors in College Students' Employability

The Pearson correlation coefficient method was used to explore CP, DP, CA, LA, AIC, AIA, AIQ, self-regulation, and college students’ employability. As can be seen from Table 4, the correlation coefficients of CP, DP, CA, LA, AIC, AIA, AIQ, and self-regulation were 0.660, 0.761, 0.799, 0.794, 0.805, 0.775, 0.835, 0.836, respectively, reaching a significant level of *P*<.001. Therefore, it can be preliminarily judged that CP, DP, CA, LA, AIC, AIA, AIQ, self-regulation, and college students’ employability have a significant positive correlation.

**Table 4:** Person correlation analysis among the variables. Correlation is significant when *P*<.001 (2-tailed).

Variable			Creativity power		Data power		Communication ability		Learning ability		Ability of individual recognition		Ability of individual acceptance		Artificial intelligence quotient		Self-regulation		College students’ employability
**Creativity power**																			
	*r*	1		0.719		0.702		0.726		0.654		0.635		0.876		0.682		0.660	
	*P* value	—^a^		<.001		<.001		<.001		<.001		<.001		<.001		<.001		<.001	
**Data power**																			
	*r*	0.719		1		0.811		0.731		0.748		0.646		0.906		0.733		0.761	
	*P* value	<.001		—		<.001		<.001		<.001		<.001		<.001		<.001		<.001	
**Communication ability**																			
	*r*	0.702		0.811		1		0.812		0.770		0.724		0.918		0.790		0.799	
	*P* value	<.001		<.001		—		<.001		<.001		<.001		<.001		<.001		<.001	
**Learning ability**																			
	*r*	0.726		0.731		0.812		1		0.773		0.748		0.906		0.805		0.794	
	*P* value	<.001		<.001		<.001		—		<.001		<.001		<.001		<.001		<.001	
**Ability of individual recognition**																			
	*r*	0.654		0.748		0.770		0.773		1		0.775		0.816		0.927		0.805	
	*P* value	<.001		<.001		<.001		<.001		—		<.001		<.001		<.001		<.001	
**Ability of individual acceptance**																			
	*r*	0.635		0.646		0.724		0.748		0.775		1		.763		.955		.775	
	*P* value	<.001		<.001		<.001		<.001		<.001		—		<.001		<.001		<.001	
**Artificial intelligence quotient**																			
	*r*	0.876		0.906		0.918		0.906		0.816		0.763		1		0.834		0.835	
	*P* value	<.001		<.001		<.001		<.001		<.001		<.001		—		<.001		<.001	
**Self-regulation**																			
	*r*	0.682		0.733		0.790		0.805		0.927		0.955		0.834		1		0.836	
	*P* value	<.001		<.001		<.001		<.001		<.001		<.001		<.001		—		<.001	
**College students’ employability**																			
	*r*	0.660		0.761		0.799		0.794		0.805		0.775		0.835		0.836		1	
	*P* value	<.001		<.001		<.001		<.001		<.001		<.001		<.001		<.001		—	

^a^Not applicable.

### The Hierarchical Regression Models

According to the results of the univariate analysis, we established hierarchical regression models (Table 5). Model 1 showed that AIQ was related to college students’ employability. Another variable, self-regulation ability (model 2) improved model 1, further enhanced by adding demographic variables (model 3). Additionally, 76.5% of the variance was explained by model 3 for predicting college students’ employability scores. College students’ employability scores were also positively correlated with AIQ, self-regulation ability, and students’ home location and negatively correlated with school level. Furthermore, men scored higher on college students’ employability compared with women.

**Table 5:** The hierarchical regression models with college students’ employability as the dependent variable.

Predictors			College students’ employability (N=1788)										
			B^a^		SE		β^b^		*t* test (df)		*P* value		95% CI
**Model 1 (adjusted *R^2^*=0.697; *F*_1,1787_=4113.486)**													
	AIQ^c^	1.034		0.016		0.835		64.136 (1786)		<.001		1.003 to 1.066	
**Model 2 (adjusted *R^2^*=0.761; *F*_2,1785_=2850.769)**													
	AIQ	0.560		0.026		0.452		21.574 (1785)		<.001		0.509 to 0.611	
	Self-regulation ability	0.928		0.042		0.460		21.942 (1785)		<.001		0.845 to 1.011	
**Model 3 (adjusted *R^2^*=0.765; *F*_7,1780_=830.355)**													
	AIQ	0.558		0.026		0.451		21.584 (1780)		<.001		0.508 to 0.609	
	Self-regulation ability	0.932		0.042		0.461		22.145 (1780)		<.001		0.849 to 1.014	
	Gender	–0.781		0.369		–0.024		–2.116 (1780)		.04		1.505 to –0.057	
	Age	–0.145		0.286		–0.006		–0.508 (1780)		.61		–0.706 to 0.416	
	School level	–1.298		0.441		–0.072		–2.941 (1780)		.003		–2.163 to –0.432	
	Degree	0.374		0.459		0.02		0.816 (1780)		.42		–0.526 to 1.274	
	Location of home	0.389		0.16		0.028		2.436 (1780)		.02		0.076 to 0.702	

^a^B: unstandardized regression weight.

^b^β: standardized regression weight.

^c^AIQ: artificial intelligence quotient.

## Discussion

### Principal Findings

Using a sample of Chinese nursing students, we found that their knowledge and use of ChatGPT are still relatively low, and their AIQ is intermediate. Additionally, this study explored associations between AIQ, self-regulation, students’ characteristics, and college students’ employability. Notably, we found that college students’ employability was significantly correlated with AIQ, self-regulation, gender, school level, and home location.

#### Chinese Nursing Students' Cognition and Demand for ChatGPT and AI

In our study, only 18.70% (335/1788) of students had used ChatGPT, and 34.60% (618/1788) had heard of ChatGPT before this survey. The rates in our study were lower than those in a study by Hosseini [27], in which 40% of participants had tried ChatGPT. These values were substantially lower than the rates in the United States, where 89% of students have used ChatGPT [28]. In our study, the most frequent reason cited for not using ChatGPT was not knowing how to access it (485/1453, 33.38%). Currently, there are still many restrictions on the OpenAI website. Due to network and registration restrictions, many Chinese students still face challenges in successfully registering for an OpenAI account to use ChatGPT. This may be one reason for the low use of ChatGPT by medical students in China. However, a survey conducted in Japan, where ChatGPT is available for use, showed that although 32% of college students had used ChatGPT, only 21.2% of medical students had used it [29]. It is worth considering whether medical students were less sensitive to emerging intelligent technologies and lacked corresponding information literacy. Previous studies have also shown that nursing students’ information literacy ability was low [30]. Thus, a general lack of consensus exists on nursing informatics ability (including information literacy) in nursing education. Additionally, nursing students receive insufficient training in nursing schools, resulting in limited proficiency in almost all areas of informatics [31,32]. However, the students in our study were very receptive to ChatGPT. Only 2.52% (45/1788) of the students said they did not accept ChatGPT, and only 6.57% (22/335) of those who had used ChatGPT said they were dissatisfied. This is similar to a previous study [33], which showed that most health care professionals had a positive or neutral attitude toward ChatGPT. ChatGPT has been very popular as a powerful AI tool since its launch. It was also considered to be bound to have an impact on industries, such as health care and education [34,35]. Therefore, developing corresponding courses and formulating relevant programs to help nursing students improve information literacy and flexibly use emerging technologies deserve further discussion.

Additionally, we found that Chinese nursing students’ AIQ was at an intermediate level, with the lowest score being in data power, consistent with the findings of previous studies [36,37]. Of note, information technology education in China started late [38]. In medical schools, where teachers’ teaching focus may be more on theoretical and operational teaching while neglecting the cultivation of students’ information ability, many schools do not offer related courses [39]. The results of this study showed that students had a positive attitude toward the use of intelligent new technologies. How to help students obtain access to technology and guide them to use technology correctly is also worthy of consideration by educators. In 2020, in “Guiding Opinions on Accelerating the Innovation and Development of Medical Education,” issued by the General Office of the State Council of China, it was pointed out that it is necessary to strengthen the deep integration of modern information technology and medical education and teaching and to explore new forms of intelligent medical education [40]. Relevant departments should respond to the development of the information age and the AI era, strengthening the training of nursing students’ information skills and AI application capabilities. Developing a training system and training program suitable for China’s national conditions and corresponding evaluation tools is also necessary. In addition, relevant courses and training can be offered, and teaching lectures can be organized to help nursing students understand the latest technology and knowledge to enhance their relevant abilities and promote the future development of medical and nursing care.

#### College Students’ Employability of Nursing Students Highly Correlated With AIQ

The results of our study showed that college students’ employability was positively correlated with the scores of AIQ, CP, DP, CA, and LA (*r*=0.660-0.835; *P*<.001). That means the higher the AIQ, the higher the employability of college nursing students. Notably, this was consistent with the findings of a previous study [41]. Students with higher AIQ had more access to new professional knowledge and advances through the internet and AI; they were aware of new developments in their disciplines and better understood the types of health care professionals in demand today [42]. Additionally, students with high AIQ tended to have stronger creativity power, communication abilities, and learning abilities. They were also better able to adapt to the employment environment and quickly completed the transition from school to the workplace [37]. Therefore, the employability of nursing college students can be enhanced by strengthening the AIQ of nursing college students, improving their ability to acquire new technologies and use them, and invoking their motivation to learn and use resources to better adapt to the environment, the workplace, and the era.

#### College Students’ Employability of Nursing Students Associated With a Variety of Factors

Our findings suggested that college students’ employability scores were positively correlated with AIQ, self-regulation ability, and students’ home location. This was consistent with the findings of previous studies [17,29-31]. Students with high self-regulation skills tend to demonstrate heightened problem awareness, individual adaptability, increased motivation in their work, and a better ability to adapt to change [43,44]. College students’ problem awareness and self-regulatory behaviors can also impact their employability. College students who are good at regulating themselves to accept new things are more likely to achieve high-quality employment in the smart era [20]. Students from urban areas may also be exposed to more employment resources and may have more opportunities for job training compared to students from rural areas [45,46]. Moreover, students from urban areas may have higher employment self-confidence and self-efficacy [47,48], which, to some extent, contributes to their higher employability compared to students from rural areas. We also found that college students’ employability scores negatively correlated with school level. This was inconsistent with a study conducted in Shanghai, China [49], which showed that College students from 211 universities have higher employability than students from ordinary colleges. The reason may be that high-level colleges such as “985” and “211” in China may pay more attention to cultivating students’ research ability, and compared to ordinary undergraduate and vocational institutions, they tend to cultivate research-oriented talents [50]. In contrast, the education of vocational institutions may focus more on cultivating technical talents [51]. Different training methods lead to differences in students’ employability in institutions of different levels [52]. Consistent with previous studies’ findings, men scored higher on college students’ employability compared to women [44]. One possible reason may be that men had the physiological advantage of having more strength and endurance [53], and psychologically, men generally demonstrated greater rational thinking abilities [54]. Nursing is a special occupation, and the above-mentioned characteristics of men may provide advantages in adapting to changes in the nursing profession. It is also worth considering whether men are born with such advantages, or if there are certain differences in the education of different genders, potentially resulting in men receiving more strength training and being more familiar with electronic technology. Thus, in terms of future education, it should be considered whether women can receive such training to enhance their adaptability in the nursing profession.

In summary, schools, governments, and educators need to pay attention to the cultivation and development of AIQ and self-regulation abilities to enhance nursing students’ employability. Therefore, conducting teaching lectures as well as setting up related courses and training can be considered to improve these training aspects. In addition, more attention and assistance should be given to students from rural areas and female students, for whom information competency training and career planning training courses can be considered. Furthermore, more attention should be paid to high-level talents from key colleges (eg, 985 and 211) to enhance their employability, cultivating comprehensive and versatile talents.

### Strengths and Limitations

At present, there are few awareness and demand surveys on ChatGPT in China. At the same time, less attention is paid to the AIQ and the employability of nursing students. Thus, this study enriches research in this field.

We collected 1788 samples from 30 provincial-level administrative regions in China through a web-based questionnaire and snowball sampling. However, since these are samples of convenience, they may not be a fully representative sample.

Although we appealed to nursing students from all regions on the internet to participate in this survey, we did not receive samples from several administrative regions. Additionally, the sample size varied significantly between administrative regions. For example, more than half of the respondents were from the Hunan Province; therefore, there may be some geographical bias. In the future, broader investigations should be considered.

### Conclusions

For Chinese nursing students, the cognition and use of ChatGPT are still relatively low, and their AIQ is at an intermediate level. Additionally, the college students’ employability was significantly correlated with AIQ, self-regulation, gender, school level, and home location. Therefore, for future education, we should pay attention to the cultivation and development of AIQ and Chinese nursing students’ self-regulation abilities. Furthermore, the employability of students from rural areas, female students, and key college students deserves more attention and guidance.

## Abbreviations

AI: artificial intelligence
AIA: ability of individual acceptance
AIC: ability of individual recognition
AIQ: artificial intelligence quotient
CA: communication ability
CP: creativity power
DP: data power
LA: learning ability

## Data Availability

The data sets generated and analyzed during this study are not publicly available due to our privacy principles for the participants but are available from the corresponding author upon reasonable request.
